# Coenzyme Q Metabolism Is Disturbed in High Fat Diet-Induced Non Alcoholic Fatty Liver Disease in Rats

**DOI:** 10.3390/ijms13021644

**Published:** 2012-02-02

**Authors:** Elena Bravo, Simonetta Palleschi, Barbara Rossi, Mariarosaria Napolitano, Luca Tiano, Emanuela D’Amore, Kathleen M Botham

**Affiliations:** 1 Department of Cell Biology and Neuroscience, National Institute of Health, Rome 00161, Italy; E-Mail: mariarosaria.napolitano@iss.it; 2 Department of Hematology, Oncology and Molecular Medicine, National Institute of Health, Rome 00161, Italy. E-Mails: simonetta.palleschi@iss.it (S.P.); barbara.rossi@iss.it (B.R.); 3 Department of Biochemistry, Biology & Genetics, Marche Polytechnic University, Ancona 00161, Italy; E-Mail: luca.tiano@unicam.it; 4 Service for Biotechnology and Animal Welfare, Animal Experimentation Sector, National Institute of Health, Rome 00161, Italy; E-Mail: emanuela.damore@iss.it; 5 Department of Veterinary Basic Sciences, the Royal Veterinary College, Royal College St., London NW1 0TU, UK; E-Mail: kbotham@rvc.ac.uk

**Keywords:** antioxidants, oxidative stress, non alcoholic fatty liver disease, Coenzyme Q, protein thiol groups, rats

## Abstract

Oxidative stress is believed to be a major contributory factor in the development of non alcoholic fatty liver disease (NAFLD), the most common liver disorder worldwide. In this study, the effects of high fat diet-induced NAFLD on Coenzyme Q (CoQ) metabolism and plasma oxidative stress markers in rats were investigated. Rats were fed a standard low fat diet (control) or a high fat diet (57% metabolizable energy as fat) for 18 weeks. The concentrations of total (reduced + oxidized) CoQ9 were increased by >2 fold in the plasma of animals fed the high fat diet, while those of total CoQ10 were unchanged. Reduced CoQ levels were raised, but oxidized CoQ levels were not, thus the proportion in the reduced form was increased by about 75%. A higher percentage of plasma CoQ9 as compared to CoQ10 was in the reduced form in both control and high fat fed rats. Plasma protein thiol (SH) levels were decreased in the high fat-fed rats as compared to the control group, but concentrations of lipid hydroperoxides and low density lipoprotein (LDL) conjugated dienes were unchanged. These results indicate that high fat diet-induced NAFLD in rats is associated with altered CoQ metabolism and increased protein, but not lipid, oxidative stress.

## 1. Introduction

Non alcoholic fatty liver disease (NAFLD) is characterised by the accumulation of triacylglycerol (TG) inside liver cells. It is relatively common in the general population, with an estimated occurrence of 20–30%, but its incidence is greatly increased to 70–90% in obesity, type 2 diabetes and related metabolic diseases and it is considered to be the hepatic manifestation of the metabolic syndrome [1]. Although the simple hepatic steatosis seen in NAFLD is thought to be relatively benign, the condition sometimes progresses into more serious liver disease, including non-alcoholic steatohepatitis (NASH), liver fibrosis, cirrhosis, and in a few cases, hepatocellular carcinoma and liver failure [1]. Progression of the disease is more likely to occur in patients with metabolic diseases [2], but the factors involved are not well understood. Both insulin resistance and oxidative stress, however, are believed to play important roles [3].

The disturbances in liver lipid metabolism that lead to NAFLD are believed to follow from the development of insulin resistance, with the steatosis then in turn exacerbating the insulin resistance [1]. Studies showing that the severity of fat deposition in the liver is a good predictor of the likelihood of progression of NAFLD to NASH led to the proposal of the ‘two hit’ hypothesis [4]. The accumulation of fat in the tissue was proposed to be the first hit which then increased the sensitivity of the liver to the second hits, mainly from increased oxidative stress and the release of pro-inflammatory cytokines, which cause the inflammation and tissue damage [4,5]. Since the hypothesis was formulated, a great deal of evidence has accumulated to support a role for oxidative stress in NAFLD [3,6]. Insulin resistance leads to increased oxidation in the liver, raised production of reactive oxygen species, higher levels of hepatic lipid peroxidation, protein oxidation and pro-inflammatory cytokine production [3,7–9] and decreased antioxidant capacity in the plasma [10]. In contrast, the importance of steatosis as the first hit is now increasingly questioned in the light of recent studies indicating that oxidative stress and the other proposed second hits all also cause hepatic fat deposition [11,12]. Thus, oxidative stress is now viewed as one of the major causes of NAFLD.

Naturally occurring lipid soluble antioxidants include carotenoids, vitamins A and E and Coenzyme Q (CoQ) (also called ubiquinone). Of these, CoQ is the only one that is synthesised endogenously in animals. It is produced in all tissues and cells, and in the inner mitochondrial membrane it acts as a redox carrier in the respiratory chain [13]. It is present, however, in all intracellular membranes and in plasma, and reduced CoQ is believed to function as an antioxidant to protect lipids from peroxidation [13]. Mitochondrial dysfunction is a feature of NAFLD [14], and a recent study in our laboratory has suggested that body concentrations of CoQ may be disturbed in the condition [15].

Animal studies of NAFLD have commonly used rodents with a genetic defect or fed a diet deficient in choline or methionine [16]. In humans, however, the condition is usually associated with caloric overconsumption, and methods have now been developed to induce NAFLD in animals in a similar way [4,17]. Our previous work has shown that a diet containing 57% of energy from fat induces the characteristic features of NAFLD in rats, including insulin resistance, hypertriglyceridemia, hepatic steatosis and liver damage, and thus provides a suitable model for the early stages of the disease [15,18]. In the present study, we have used this model to test the hypothesis that CoQ metabolism is disturbed in NAFLD, using our previously characterized high fat diet-induced rat model of the disease. In addition, we have studied the effects of NAFLD on other oxidative stress markers including protein SH, the antioxidants glutathione (GSH) and vitamins A (VitA) and E (VitE) in blood.

## 2. Results and Discussion

### 2.1. Plasma Lipids, Enzymes, Insulin and Homeostatic Model Assessment of Insulin Resistance (HOMA-IR)

**Figure 1:**
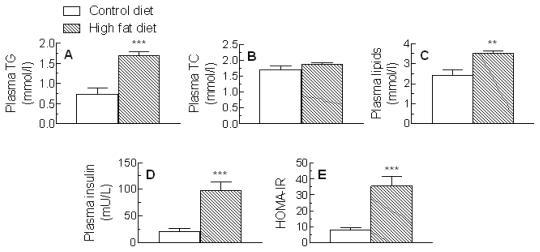
Rats were fed standard low fat diet (Control diet) or a high fat diet for 18 weeks. Blood samples were then collected and the concentrations of triacylglycerol (TG) (**A**) and total cholesterol (TC) (**B**), total lipids (TG + TC) (**C**) and insulin (**D**) in plasma were determined and the Homeostatic model assessment of insulin resistance (HOMA-IR) (**E**) was calculated. Data are the mean from 7 (Control diet) or 10 (High fat diet) animals and error bars show the SEM. ** *P* < 0.01, *** *P* < 0.001 *vs.* Control diet.

The concentrations of TG and cholesterol in plasma from rats given the control or high fat diet are shown in Figure 1. As expected, plasma TG levels were markedly increased by the high fat diet (Figure 1A), but plasma cholesterol concentrations were unchanged (Figure 1B). Total plasma lipids (TG + cholesterol) were raised by about 30% (Figure 1C).

Increased plasma alanine aminotransferase (ALT) activity is considered to be a biomarker of NAFLD and a predictor of disease progression [19] and an AST/ALT ratio <1 indicates that NAFLD is present [20]. In our experiments, plasma activity of ALT was elevated by >3 fold (*P* < 0.01) in high fat-fed as compared to control rats, while aspartate animotransferase (AST) activity was unchanged. Thus, the AST:ALT ratio was decreased by about 60% after high fat diet feeding (Table 1). Plasma insulin levels were about 4.5 fold higher in rats fed the high fat diet than in those fed the control diet (Figure 1D) and the HOMA-IR was also raised by about 4.5 fold (Figure 1E). There was no significant change, however, in plasma glucose levels (Table 1).

### 2.2. Liver Lipids and Histology

Liver TG concentrations were increased by 3.1 fold in the high fat diet as compared to the control diet group (control diet, 18.3 ± 4.3 mg/g liver, *n* = 7; high fat diet 56.8 ± 3.8 mg/g liver, *n* = 8, *P* < 0.001), while liver cholesterol levels were raised by 29% (control diet 32.3 ± 1.0 mg/g liver, *n* = 7; high fat diet 41.7 ± 1.9 mg/g liver, *n* = 10, *P* < 0.01).

**Figure 2:**
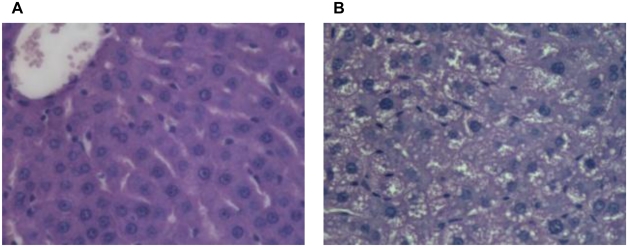
Rats were fed standard low fat diet (Control diet) or a high fat diet for 18 weeks. Livers were collected and sections were stained with haematoxylin-eosin (H&E). (**A**) Control diet; (**B**) High fat diet. Images typical of sections taken from 7 rats fed the control diet and 10 rats fed the high fat diet are shown. Original magnification 40×.

Liver sections from rats fed the control diet showed no visible accumulation of lipid (Figure 2A), but in sections obtained from animals fed the high fat diet many lipid droplets could be seen inside hepatocytes, and this sometimes caused the nucleus to be displaced to the periphery of the cells.

These histological changes, together with the disturbances in plasma ALT activity, ALT/AST ratio, insulin levels and the HOMA-IR indicate that the high fat diet-fed animals have developed a fatty liver, and are in agreement with our previous comprehensive characterisation of the high fat diet as a model for the induction of NAFLD [15].

### 2.3. Coenzyme Q

**Figure 3:**
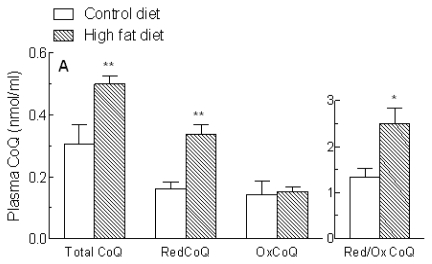

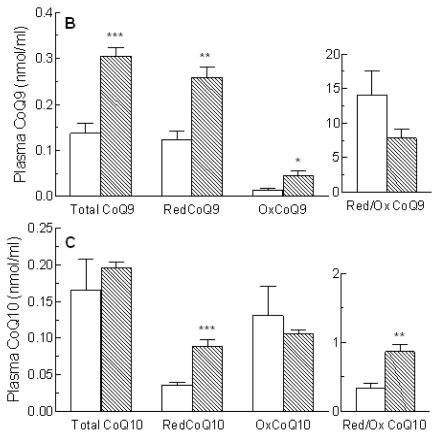
Plasma Coenzyme Q (CoQ) levels in rats fed a standard low fat (Control diet) or a high fat diet for 18 weeks. Blood samples were collected and the concentrations of reduced (RedCoQ) and oxidised (OxCoQ) CoQ9 and CoQ10 in plasma were determined. Total CoQ values are RedCoQ + OxCoQ. (**A**) CoQ (CoQ9 + CoQ10) and RedCoQ/OxCoQ; (**B**) CoQ9 and RedCoQ9/OxCoQ9; (**C**) CoQ10 and RedCoQ10/ OxCoQ10; Data are the mean from 6 (Control diet) or 10 (High fat diet) animals and error bars show the SEM. * *P* < 0.05, ** *P* < 0.01, *** *P* < 0.001 *vs.* Control diet.

**Figure 4:**
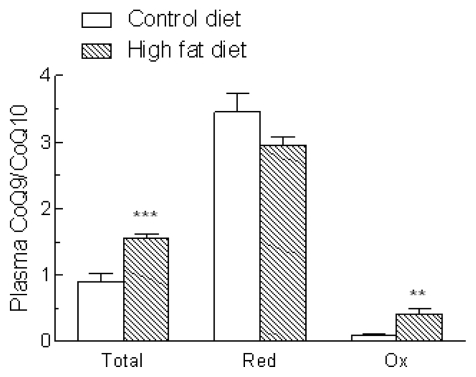
Plasma CoQ9:CoQ10 ratios in rats fed a standard low fat (Control diet) or a high fat diet for 18 weeks. Blood samples were collected and the concentrations of reduced (RedCoQ) and oxidised (OxCoQ) CoQ9 and CoQ10 in plasma were measured and the ratio of CoQ9:CoQ10 was calculated. Data are the mean from 6 (Control diet) or 10 (High fat diet) animals and error bars show the SEM. ** *P* < 0.01, *** *P* < 0.001 *vs.* Control diet.

In humans and many other mammals, the side chain of CoQ has 10 isoprene residues (CoQ10), but the major form synthesized in rats and mice has only 9 (CoQ9) [13]. Rat plasma and tissues, however, also contain some CoQ10, which originates from endogenous biosynthesis and also from exogenous sources. The concentrations of CoQ9, CoQ10 and CoQ9 + CoQ10 in the plasma of rats fed the control and high fat diets are shown in Figure 3. Overall CoQ (CoQ9 + CoQ10) levels (Figure 3A) were significantly increased in the plasma of animals fed the high fat diet (+64%, *P* < 0.01), and this was entirely due to a >2 fold rise in CoQ9 (Figure 3B), as CoQ10 concentrations (Figure 3C) were not significantly changed. Thus, the ratio of CoQ9:CoQ10 increased from 0.9 to 1.6 (Figure 4). Levels of reduced, but not oxidized, CoQ9 + CoQ10 were also markedly raised, and as a consequence there was an increase of about 75% in the proportion of CoQ9 + CoQ10 found in the reduced form (Figure 3A).

Most of the CoQ9 in the plasma of the control rats was in the reduced form (reduced:oxidized ratio about 14), and the proportions were not significantly changed by high fat feeding, since the levels of both reduced and oxidized CoQ9 were increased (Figure 3B). In contrast, in the control group plasma CoQ10 was mainly in the oxidized form (reduced:oxidized ratio, 0.34). Reduced CoQ10 concentrations were increased about 2 fold after high fat feeding, but oxidized CoQ10 levels remained unchanged, thus the reduced:oxidized ratio (0.87) was significantly increased. However, it remained markedly lower than that of CoQ9 (Figure 3C). The ratio of CoQ9:CoQ10 (Figure 4) was 3–3.5 for the reduced form regardless of the diet, but for oxidized CoQ it was increased from 0.1 in the control group to 0.4 after high fat feeding, because of the increase in oxidized CoQ9 (Figure 3B).

### 2.4. Blood Antioxidants and Oxidative Stress Markers

**Figure 5:**
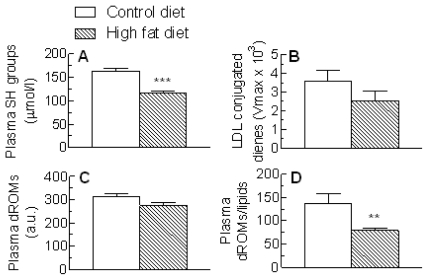
Blood oxidative stress markers in rats fed a standard low fat (Control diet) or a high fat diet for 18 weeks. Blood samples were collected and the plasma or LDL (conjugated dienes formation in response to copper oxidation) content of (**A**) protein **thiol (**SH) groups; (**B**) conjugated dienes (*V*_max_ × 10^3^ of propagation phase, determined using 50 μg/mL LDL protein); (**C**) reactive oxygen species (dROMs) was determined. (**D**) dROMs/plasma lipids. Data are the mean from 6 (control diet) or 9 (High fat diet) animals (protein SH groups and dROMs). For conjugated dienes the mean of 5 rats in each group are shown. Error bars show the SEM. ** *P* < 0.01, *** *P* < 0.001 *vs.* Control diet.

Levels of protein thiol (SH) groups in the plasma were significantly reduced in the high fat-fed as compared to control rats (*P* < 0.001) (Figure 5A). LDL conjugated diene formation in response to copper oxidation, however, was not significantly changed (Figure 5B). The absolute reactive oxygen species (dROM) values were similar in animals given the control and high fat diets (Figure 5C), but when the difference in lipid concentrations after high fat feeding (Figure 1) was taken into account, there was a significant reduction in the high fat-fed group (*P* < 0.01) (Figure 5D).

**Figure 6:**
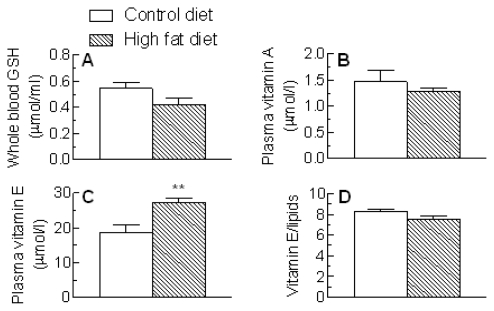
Plasma antioxidant levels in rats fed a standard low fat (Control diet) or a high fat diet for 18 weeks. Blood samples were collected and the concentrations of (**A**) Red blood cell glutathione (GSH) (data expressed as μmol/mL whole blood); (**B**) VitA; and (**C**) VitE were determined. (**D**) VitE/plasma lipids (μmol/mmol). Data for GSH are the mean from 7 (Control diet) or 9 (High fat diet) animals and those for vitamins A and E are the mean from 4 (control diet) or 6 (High fat diet) rats. Error bars show the SEM. ** *P* < 0.01 *vs.* Control diet.

The effects of the high fat diet on other plasma antioxidant levels are shown in Figure 6. Red blood cell GSH and plasma VitA concentrations were not significantly changed after feeding the high fat diet (Figure 6A,B), but VitE levels were increased (*P* < 0.01) (Figure 6C). This change, however, appeared to be related to the increase in plasma lipid concentrations in the high fat group (Figure 1), since the VitE: total lipid ratio was unaffected (Figure 6D).

In recent years, oxidative stress has emerged as an important factor in the development of NAFLD [3,6] and it is now thought to be a cause as well as a result of hepatic steatosis [7,19,21]. In this study, we investigated changes which occur in plasma oxidative stress markers, particularly Coenzyme Q (CoQ), and other antioxidants in high fat-diet induced model of NAFLD in rats. The results showed that NAFLD causes a marked rise in the plasma content of the antioxidant CoQ which is accompanied by an increase in the ratio of the reduced to the oxidized form, and is also associated with increased protein oxidative stress in the plasma aqueous compartment. Plasma lipid peroxidation and antioxidant capacity as well as LDL propensity to oxidation, however, remain unchanged.

Markedly increased concentrations of CoQ9 (the main form synthesized in rats *in vivo*) in the plasma and liver mitochondria have been reported in rat models of both type 1 and type 2 diabetes [22,23] and in a mouse model of NASH [24]. This rise in endogenous synthesis is believed to be an adaptation to systemic oxidative stress [22,25,26]. Peroxisome proliferation in the liver in rats occurs in diabetes and in response to feeding high fat diets, and is known to induce CoQ synthesis without affecting breakdown [22,23,25]. Enhanced lipid peroxidation related to physical exercise and dietary factors has also been reported to lead to increased tissue levels of CoQ in rats [27].

In the present study, we found a marked rise in CoQ (CoQ9 + CoQ10) in the plasma of the high fat diet-induced NAFLD rats (+64%), which was caused by an increase in the level of CoQ9 (Figure 3A), and thus resulted in a higher ratio of CoQ9:CoQ10 (Figure 4). The ratio of reduced:oxidized CoQ9 was unchanged by high fat feeding as concentrations of both forms were raised (Figure 3B), but for CoQ10 the high fat diet increased only the reduced form, thus the reduced: oxidized CoQ10 ratio was higher in the high fat-fed compared to control rats (Figure 3C), and consequently there was an increase in the overall reduced:oxidized ratio of CoQ (Figure 3A). Thus, in addition to a rise overall CoQ in the plasma of the high fat diet-induced NAFLD rats, the proportion of reduced CoQ is also increased. It is known that reduced CoQ is the most effective antioxidant in cell membranes [13], where it protects phospholipids and membrane proteins from free radical-induced oxidative damage. In addition, it helps to prevent lipid peroxidation in lipoproteins in plasma [25]. Reduced and oxidized CoQ are interconvertible in the body, as the quinone/quinol group can either accept or donate electrons, and the antioxidant effects of the reduced form are independent of those of antioxidants of dietary origin [13]. Since the rise in plasma total CoQ induced by the high fat diet in the present study was due to a >2 fold increase in CoQ9, the main form synthesized endogenously in rats, while levels of CoQ10, the form found in animal fats such as lard (the fat supplement in the high fat diet used here), were unchanged (Figures 3B,C and 4), the observed increase is likely to be due to an increase in endogenous CoQ synthesis, rather than increased intake in the diet. Since it is known that oxidative stress caused by insulin resistance and fat accumulation in the liver is associated with NAFLD development, our findings suggest that this triggers increased endogenous synthesis of CoQ9 and also a rise in the proportion in the reduced state. This adaptive response may then help to protect against a reduction in the antioxidant capacity of the plasma and against lipid peroxidation in the model of the early stages of the disease used here [15].

Plasma levels of a range of oxidative stress markers and antioxidants in rats with high fat diet-induced NAFLD were also determined in the present study. Plasma concentrations of the oxidative stress marker, hydroxalkenal, have been reported to be increased when rats were fed a high fat diet [28], however, other workers found no change in the levels of F2-isoprostanes after high fat-feeding [29]. In our experiments, there was a clear decrease of about 30% in protein SH groups in the plasma of the high fat-fed rats, indicating increased protein oxidative stress in the plasma aqueous compartment (Figure 5A). Plasma conjugated dienes and dROM, however, were not significantly changed (Figure 5B) and in fact dROM were markedly reduced (−43%, Figure 5D) when the rise in plasma lipids (Figure 1) was taken into account, indicating that lipid peroxidation was not increased by the high fat diet. The lack of effect of the high fat diet on lipid peroxidation, even though protein oxidative stress was increased, may be partly explained by the use of saturated fat as the fat supplement. However, it may also be related to changes in the metabolism of the antioxidant CoQ, as discussed above.

We also found no evidence for decreased plasma antioxidant levels in the high fat-fed rats. Red blood cell GSH and plasma VitA concentrations were not significantly changed (Figure 6A,B) and plasma VitE levels were increased, rather than decreased, although this was related to the increased plasma lipid levels (Figure 6C,D). The different effects of the high fat diet on blood concentrations of the two lipophilic vitamins may be explained by their different carriers. VitE is associated with the plasma lipoproteins, thus the rise in its levels disappears when corrected for the increase in plasma lipids, but most VitA in blood is bound to retinol binding protein, and our results suggest that concentrations of this protein remain unchanged.

## 3. Experimental Section

### 3.1. Animals and Diets

Male Wistar rats weighing approximately 200 g (6 weeks old) were obtained from Harlan, Italy (S. Pietro al Natisone, Italy), housed individually at a temperature of 22 °C with a 12-h light-dark cycle and allowed food and water *ad libitum*. The studies were carried out in accordance with the guidelines of the European Community Council for animal care and use, and all procedures conformed strictly to Decree 116/92, which represents the Italian enforcement of the European Directive 86/609/ EEC.

The experimental protocol was approved by the Animal Care Ethics Committee of the Istituto Superiore di Sanità (ISS) and, according to the national law requirement the communication of the study has been sent by the ISS to the Animal Welfare Office (Office VI) of the Italian Minister of Health (Communication Protocol Number 983/SSA/07). The animals were divided into two groups and fed either a standard low fat rat chow diet (4.3% fat, 10% of the metabolizable energy) (control diet, 7 rats) or a diet containing 35% fat (31.6% saturated fat and 3.2% unsaturated fat, 57% of the metabolizable energy) (Mucedola srl, Settimo Milanese, Italy) (high fat diet, 10 rats) for 18 weeks. The composition of the diets was as described by Cano *et al.* [18].

At the end of the experiment all rats were sacrificed after fasting overnight. Blood samples collected in heparinized tubes via heart puncture with the rats under terminal anesthesia (Acepromazine 2.5 mg/kg + Xylazine 1–5 mg/kg im) were centrifuged (3500 rpm, 15 min, at 6 °C) to obtain the plasma. Because of the labile nature of oxidative stress biomarkers, plasma was isolated from blood samples as soon as possible after collection and aliquots were either immediately assessed (reactive oxygen metabolites, dROM, and thiol groups, SH) or stored at −30 °C prior to analysis (VitA, VitE, cholesterol, TG) or −80 °C (conjugated dienes, CoQ9, CoQ10). For GSH determination, pre-treated (see Other analytical methods, below) red blood cells were kept at −80 °C. Livers were excised, washed with cold physiological saline (0.9%), and samples (200 mg) were homogenised in methanol (5 mL). One lobe of each liver was placed in buffered formalin solution (10% w/v). For histopathological examination, samples were embedded in paraffin and sections (approximately 2 μm in thickness) were cut from each tissue block using a microtome. Sections were deparaffinized in xylene, rehydrated through a graded ethanol series and stained with haematoxylin-eosin (H&E). Histological examination was carried out by an investigator without knowledge of the treatments.

Lipids were extracted by the method of Folch *et al.* [30].

### 3.2. Determination of Conjugated Dienes

A starting sample of 0.5 mL of plasma was used for the isolation of lipoprotein fractions by step ultracentrifugation according to Friedewald *et al.* [31], adapted for fractionation of 0.5 mL plasma sample in a TLA-100.2 fixed angle rotor in a Beckmann TLA-100 Tabletop Ultracentrifuge. Low density lipoprotein (LDL) (1.006–1.063 g/mL) was dialyzed overnight against phosphate buffer solution. 50 μg LDL protein /mL was incubated with CuSO_4_ (final concentration, 10 μM) and conjugated dienes formation was assayed by measuring the absorbance at 234 nm at 30 °C for 600 min. The kinetic profile parameters of LDL oxidation calculated according to Pinchuk and Lichtenberg [32] are reported in term of *V*_max_ (slope of the curve in the propagation phase) (OD/min units).

### 3.3. Other Analytical Methods

The TG and cholesterol content of plasma was assayed using enzyme-based kits (BioSed S.r.l., Rome, Italy). Protein concentrations were determined using commercial bicinchoninic acid reagent (Pierce) (liver supernatants) or the method of Bradford [33]. Plasma insulin was measured by ELISA (Mercodia, Uppsala, Sweden), and fasting plasma glucose levels (BPC BioSed srl, Rome, Italy) and alanine aminotransferase (ALT) and aspartate animotransferase (AST) activities (FAR srl, Verona, Italy) were determined using commercial kits according to the manufacturers’ instructions. Plasma VitA, VitE, CoQ9, CoQ10 concentrations were evaluated by HPLC coupled with UV, fluorescence or electrochemical detection as described previously [34–36]. Total dROM and SH were evaluated using commercial photometric tests (dROM and SHp tests, respectively, Diacron International, GR, Italy). The dROM test measures lipid hydroperoxides, which are generated by the oxidative attack of ROS on various lipid substrates. In the assay, alkoxyl and peroxyl radicals produced by iron-catalyzed degradation of the plasma lipid hydroperoxides are photometrically detected after reacting (75 min at 37 °C) with a chromogenic alkylamine [37]. Values are indicated as arbitrary units (a.u.; 1 a.u. = 0.8 mg/L H_2_O_2_). The SHp test is based on the ability of SH to develop a colored complex photometrically detectable after reacting (4 min at room temperature) with 5,5′-dithiobis-2-nitrobenzoic acid (DTNB). The results are expressed as μmol SH/L.

For GSH determination, whole blood was centrifuged at 2500 g at 4 °C for 10 min; the supernatant was removed and the pellets of erythrocytes were washed once with cold phosphate buffered saline (PBS). The pellet was diluted with 4 volumes of 6% (w/v) of ice-cold metaphosphoric acid, and centrifuged at 2000 g for 10 min at 4 °C to remove the protein precipitate. The clear supernatants were then used to determine the GSH content, according to Anderson [38]. The hematocrit values did not significantly differ between the two rat groups and the data are expressed in terms of μmol/mL of whole blood.

Significance limits were calculated using Student’s *t* test with GraphPad Prism software. Changes were considered significant at *P* < 0.05.

## 4. Conclusions

Our findings indicate that high fat diet-induced NAFLD in rats leads to a marked increase in the plasma content of the antioxidant CoQ, and that this is entirely due to an increase in the levels of CoQ9, which is synthesised endogenously in rats, rather than obtained from the diet. This rise in CoQ9 synthesis is likely to be an adaptive response to the oxidative stress associated with NAFLD [22,25,26]. In addition, protein, but not lipid, oxidative stress is increased. The increase in CoQ levels may help to prevent increased lipid peroxidation and to counter a reduction in antioxidants in these animals.

## Figures and Tables

**Table 1:** Plasma glucose, alanine aminotransferase (ALT) and aspartate animotransferase (AST) levels in rats fed a standard low fat (Control diet) or a high fat diet for 18 weeks. Blood samples were collected and the concentration of glucose and the activity of ALT and AST was measured. Data are the mean ± SEM from 6 (Control diet) or 9 (High fat diet) animals.

Parameter	Control diet	High fat diet
Glucose (mmol/L)	8.75 ± 0.74	8.07 ± 0.25
ALT (U/L)	33.5 ± 4.6	111.4+19.4 **
AST (U/L)	52.5 ± 5.1	61.1 ± 3.9
AST/ALT	1.65 ± 0.17	0.66 ± 0.07 ***

** *P* < 0.01,

*** *P* < 0.001 *vs.* Control diet.
